# Individualized brain development and cognitive outcome in infants with congenital heart disease

**DOI:** 10.1093/braincomms/fcab046

**Published:** 2021-03-23

**Authors:** Alexandra F Bonthrone, Ralica Dimitrova, Andrew Chew, Christopher J Kelly, Lucilio Cordero-Grande, Olivia Carney, Alexia Egloff, Emer Hughes, Katy Vecchiato, John Simpson, Joseph V Hajnal, Kuberan Pushparajah, Suresh Victor, Chiara Nosarti, Mary A Rutherford, A David Edwards, Jonathan O’Muircheartaigh, Serena J Counsell

**Affiliations:** 1 Centre for the Developing Brain, School of Biomedical Engineering and Imaging Sciences, King’s College London, London SE1 7EH, UK; 2 Department for Forensic and Neurodevelopmental Sciences, Institute of Psychiatry, Psychology and Neuroscience, King’s College London, London SE5 8AF, UK; 3 Biomedical Image Technologies, ETSI Telecomunicación, Universidad Politécnica de Madrid and CIBER-BBN, 28040 Madrid, Spain; 4 Paediatric Cardiology Department, Evelina London Children’s Healthcare, London SE1 7EH, UK; 5 Department of Child and Adolescent Psychiatry, Institute of Psychiatry, Psychology and Neuroscience, King's College London, London SE5 8AF, UK

**Keywords:** congenital heart disease, brain, cognition, MRI, dHCP

## Abstract

Infants with congenital heart disease are at risk of neurodevelopmental impairments, the origins of which are currently unclear. This study aimed to characterize the relationship between neonatal brain development, cerebral oxygen delivery and neurodevelopmental outcome in infants with congenital heart disease. A cohort of infants with serious or critical congenital heart disease (*N* = 66; *N* = 62 born ≥37 weeks) underwent brain MRI before surgery on a 3T scanner situated on the neonatal unit. T2-weighted images were segmented into brain regions using a neonatal-specific algorithm. We generated normative curves of typical volumetric brain development using a data-driven technique applied to 219 healthy infants from the Developing Human Connectome Project (dHCP). Atypicality indices, representing the degree of positive or negative deviation of a regional volume from the normative mean for a given gestational age, sex and postnatal age, were calculated for each infant with congenital heart disease. Phase contrast angiography was acquired in 53 infants with congenital heart disease and cerebral oxygen delivery was calculated. Cognitive and motor abilities were assessed at 22 months (*N* = 46) using the *Bayley scales of Infant and Toddler Development*–Third Edition. We assessed the relationship between atypicality indices, cerebral oxygen delivery and cognitive and motor outcome. Additionally, we examined whether cerebral oxygen delivery was associated with neurodevelopmental outcome through the mediating effect of brain volume. Negative atypicality indices in deep grey matter were associated with both reduced neonatal cerebral oxygen delivery and poorer cognitive abilities at 22 months across the whole sample. In infants with congenital heart disease born ≥37 weeks, negative cortical grey matter and total tissue volume atypicality indices, in addition to deep grey matter structures, were associated with poorer cognition. There was a significant indirect relationship between cerebral oxygen delivery and cognition through the mediating effect of negative deep grey matter atypicality indices across the whole sample. In infants born ≥37 weeks, cortical grey matter and total tissue volume atypicality indices were also mediators of this relationship. In summary, lower cognitive abilities in toddlers with congenital heart disease were associated with smaller grey matter volumes before cardiac surgery. The aetiology of poor cognition may encompass poor cerebral oxygen delivery leading to impaired grey matter growth. Interventions to improve cerebral oxygen delivery may promote early brain growth and improve cognitive outcomes in infants with congenital heart disease.

## Teaser

Bonthrone et al. assessed individualized brain growth in infants with congenital heart disease compared with a large normative sample to determine atypicality indices in the patient group. Low cerebral oxygen delivery was associated with impaired grey matter development and resulted in poor cognitive outcome in this population.

## Graphical Abstract

**Graphical Abstract:**
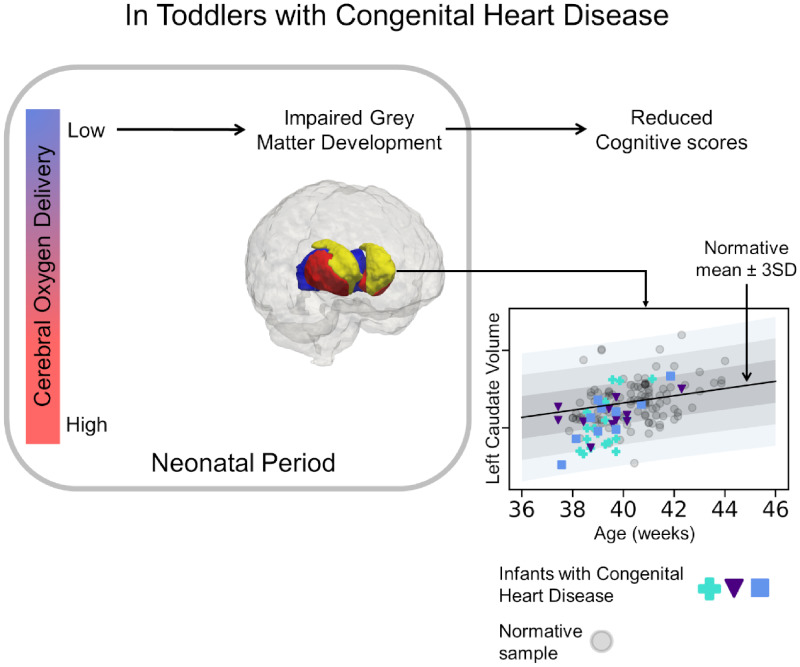

## Introduction

Congenital heart disease (CHD) is the most common congenital malformation, occurring in up to 1% of births.^1^ Increasing numbers of infants survive into adulthood,^2^ yet up to half of the survivors of CHD will show developmental impairments across several domains including executive functioning, speech and language, motor coordination and cognition.^3^^,^^4^ These deficits may persist into adolescence^5^ and adulthood^6^ and have a prolonged impact on the quality of life^7^ and education.^8^ As such, understanding the mechanisms underpinning developmental disabilities in survivors of CHD is critical.

MRI studies have identified reduced brain volumes in foetuses and neonates with CHD compared with healthy controls.^9–12^ Altered early brain development is associated with reduced cerebral oxygen delivery (CDO_2_),^13–15^ and impaired CDO_2_ may contribute to poor neurodevelopmental outcome in this population.^16^ However, to our knowledge, no study has characterized the relationship between CDO_2_, neonatal brain development and subsequent neurodevelopmental outcome in infants with CHD.

The literature linking intracranial volumes to outcome in children with CHD is limited. Poorer cognitive abilities in early childhood have been associated with dilated ventricular and extracerebral CSF spaces^17^^,^^18^ and reduced cortical and cerebellar volumes^19^ on postsurgical imaging. At age six, children with CHD and IQ scores below 85 had lower postoperative neonatal basal ganglia, thalamus and brainstem volumes compared with survivors with higher IQ scores.^20^

To better understand the relationship between early brain development, before surgery and neurodevelopmental outcome in infants with CHD, an individualized assessment of cerebral development is needed. Mapping datasets from babies with CHD to robust normative neonatal data allows for the assessment of individualized neonatal brain development. Such normative modelling approaches characterize typical population variation in a data-driven fashion and have been applied to neonatal^21^^,^^22^ and psychiatric^23^ neuroimaging cohorts. These models can be used to quantify the deviation of an individual from the expected mean in the typical population, termed an ‘atypicality index’, in a process akin to the use of growth charts to track foetal and paediatric development. In contrast to case–control studies, this approach does not assume that the effect of CHD on brain development is homogenous across infants.

In this study, we calculated atypicality indices, representing the deviation of brain volumes from typical neonatal brain development, in a cohort of neonates with CHD. Normative curves were derived from a large sample of healthy term born infants using a Gaussian process regression (GPR) model.^24^ We aimed to determine both the average degree of deviation and the prevalence of extreme deviations from typical brain volumetric development in infants with CHD before surgery. We also investigated the relationship between atypicality indices, neonatal cerebral oxygen delivery and cognitive and motor scores at 22 months.

## Materials and methods

### Ethical approval

The National Research Ethics Service West London committee provided ethical approval (CHD: 07/H0707/105; dHCP: 14/LO/1169). In accordance with the declaration of Helsinki, informed written parental consent was obtained before MRI and follow-up.

### Infants with CHD

#### Recruitment

A prospective cohort of 66 infants with critical or serious CHD [39 male, median (Interquartile range IQR) gestational age at birth = 38.5 (38.1–38.9); 62 born ≥37 weeks] was recruited (Table 1). Based on a previously published UK categorization,^25^ critical CHD was defined as infants with hypoplastic left heart syndrome (HLHS), interrupted aortic arch, pulmonary atresia with an intact ventricular septum, simple transposition of the great arteries (TGA) and all infants requiring surgery within the first 28 days of life with the following conditions: Aortic valve stenosis, coarctation of the aorta (CoA), pulmonary valve stenosis, pulmonary atresia with ventricular septal defect, tetralogy of Fallot (TOF) and total anomalous pulmonary venous connection. Serious CHD was defined as any cardiac lesion not defined as critical, which requires cardiac catheterization or surgery between 1 month and 1 year of age.

**Table 1:** Summary of cohort demographic information

Primary cardiac defect, *N* (%)	All infants (*N* = 66)	Infants born ≥37 weeks (*N* = 62)
Abnormal streaming of blood		
Dextro-transposition of the great arteries	31 (47)	30 (48)
Truncus arteriosus	1 (1)	1 (2)
Left-sided cardiac lesions		
Coarctation of the aorta	13 (20)	13 (21)
Hypoplastic left heart syndrome	4 (6)	4 (6)
Aortic stenosis with coarctation of the aorta	1 (1)	1 (2)
Right-sided cardiac lesions		
Tetralogy of fallot	7 (11)	7 (11)
Pulmonary stenosis	4 (6)	4 (6)
Pulmonary atresia	3 (5)	1 (2)
Tricuspid atresia	2 (3)	1 (2)
Delivery method, *N* (%)	All infants (*N* = 66)	Infants born ≥37 weeks (*N* = 62)
Spontaneous/induced vaginal delivery	25 (38)	25 (40)
Instrumental delivery	11 (17)	11 (18)
Elective caesarean section	7 (10)	6 (10)
Emergency caesarean section	23 (35)	20 (32)
Brain Injury findings, *N* (%)	All infants (*N* = 66)	Infants born ≥37 weeks (*N* = 62)
None	39 (59)	38 (61)
Mild WMI	13 (20)	11 (18)
Moderate WMI	5 (8)	5 (8)
Severe WMI	2 (3)	1 (2)
Arterial ischemic stroke	1 (1)	1 (2)
Arterial ischemic stroke with mild WMI	2 (3)	2 (3)
Cerebellar haemorrhage	3 (5)	3 (5)
Cerebellar haemorrhage with moderate WMI	1 (1)	1 (2)
Other demographic information	All infants (*N* = 66)	Infants born ≥37 weeks (*N* = 62)
Gestational age at birth, median (IQR)	38.5 (38.1–38.9)	38.6 (38.2–38.9)
Postmenstrual age at scan, median (IQR)	39.2 (38.6–39.7)	39.3 (38.7–39.7)
Antenatal diagnosis, *N* (%)	62 (94)	58 (94)
Inborn, *N* (%)	63 (96)	59 (95)
Birth weight (g), mean (SD)	3044 (503)	3104 (455)
Birth weight z-score, mean (SD)	−0.61(1.8)	−0.58 (1.10)
Head circumference (cm), mean (SD)	33.6 (1.8)	33.8 (1.7)
Head circumference z-score, mean (SD)	−0.72 (1.46)	−0.67 (1.59)
CDO_2_ (mLO_2_/min), mean (SD)	1795(495)	1818 (495)

Infants were recruited from the Neonatal Unit at St Thomas’ Hospital, London, between 2015 and 2020. Exclusion criteria included suspected or confirmed chromosomal abnormality, previous neonatal surgery (excluding cardiac catheterization procedures), gestational age <34 weeks at birth and suspected congenital infection.

#### Clinical information

Birth weight, head circumference and delivery method were extracted from clinical notes. Birth weight and head circumference were converted to z-scores based on the UK-WHO growth centiles implemented in the GrowthCharts mobile application version 2.0.1.^26^ Infants with CHD were categorized into abnormal streaming of blood, left-sided heart lesions and right-sided heart lesions based on haemodynamic impact using the sequential segmental approach.^27^

#### Magnetic resonance imaging

MRI was performed on a Philips Achieva 3 Tesla system situated on the Neonatal Unit at St Thomas’ Hospital, London. Imaging was performed during natural sleep without sedation and pulse oximetry; respiration, temperature and electrocardiography were monitored throughout by a nurse and paediatrician experienced in neonatal MRI procedures.

Infants were scanned with a 32-channel neonatal head coil and neonatal positioning system [postmenstrual age at scan median (IQR) = 39.3(38.6–39.7) weeks].^28^ Scans included a 5-s noise ramp-up to avoid a startle response. T2-weighted multi-slice turbo spin echo scans were acquired in two stacks in sagittal and axial planes [repetition time (TR)/echo time (TE) = 12 000/156 ms; flip angle = 90°; slice thickness = 1.6 mm; slice overlap = 0.8 mm; in-plane resolution: 0.8 ×0.8 mm; SENSE factor = 2.11/2.58 (axial/sagittal)]. T2-weighted volumes were reconstructed using a dedicated algorithm to correct motion and integrate data from both acquired stacks (reconstructed voxel size = 0.5 mm^3^).^29^^,^^30^ T1-weighted volumetric magnetization prepared rapid acquisition gradient echo (MPRAGE) images were also acquired (TR/TE = 11/4.6 ms; inversion time (TI) = 713 ms; flip angle = 9°; voxel size = 0.76 × 0.76 × 0.8 mm, SENSE factor = 1.2).

Infants underwent quantitative flow imaging using velocity sensitized phase contrast angiography with a single-slice T1-weighted fast field echo sequence (field of view = 100 × 100 mm^2^; resolution = 0.6 × 0.6 × 4.0 mm; TR/TE = 6.4/4.3 ms; flip angle = 10°, repetitions = 20; maximal encoding velocity = 140 cm/s). Images were acquired in a plane perpendicular to both internal carotids and basilar arteries at the level of the sphenoid bone.^31^ Phase contrast angiography was available in 53 infants (not acquired due to infant waking *N* = 7; unsuitable for analysis *N* = 6).

#### MR image review

T1- and T2-weighted images were reported by perinatal neuroradiologists. All images were reviewed by two neuroradiologists to ensure consistency and lesions were recorded as arterial ischemic stroke, white matter injury (WMI), cerebellar haemorrhage or intraventricular haemorrhage as reported previously.^32^ WMI was classified into normal (no injury), mild (≤3 foci and all ≤2 mm), moderate (>3 and ≤10 foci or any >2 mm) or severe (>10 foci).^32^^,^^33^

Three infants with CHD had an arterial ischemic stroke. For these infants, cortical and total tissue volumes were excluded from further analysis; however, subcortical and infratentorial volumes were analysed.

#### Image segmentation

T2-weighted images were processed using the dHCP structural pipeline.^34^ Images underwent bias correction and brain extraction before being segmented into eight tissue classes (cortical grey matter, white matter, total deep grey matter, cerebellum, brainstem, hippocampus and amygdala, ventricles and extracerebral CSF) with an automatic neonatal-specific segmentation algorithm that uses expectation maximization.^34^^,^^35^ Deep grey matter was further segmented into left/right: lentiform, caudate nucleus and thalamus. Segmentations were visually inspected, and minor inaccuracies were manually corrected. Regional tissue volumes were extracted. Total tissue volume (TTV) was calculated by summing cortical grey matter, white matter, cerebellum, brainstem, total deep grey matter and hippocampus and amygdala.

#### Cerebral oxygen delivery

Cerebral blood flow was quantified in infants with CHD from phase-contrast angiography using previously published methods.^14^^,^^15^ Briefly, regions of interest were drawn manually around the left/right internal carotid and basilar arteries using Segment v2.0 R4800.^36^ Flow was extracted and summed across the vessels to estimate total cerebral blood flow. Haemoglobin measurements were extracted from clinical notes at a median (IQR) of 2(1–4) days before the date of scan. Pre-ductal oxygen saturation was measured at the time of scan with a Masimo Radical‐7 monitor (Masimo Corp, Irvine, CA) applied to the right hand.

Cerebral oxygen delivery (CDO_2_) was calculated as follows^37^:

$${\text{CDO}}_{2}\left({\text{mLO}}_{2}/\text{min}\right)=\text{oxygen}\;\text{saturation}\times \text{haemoglobin}\left(g/\text{dL}\right)\times 1.36\times \text{cerebral}\;\text{blood}\;\text{flow}\;\left(\text{mL}/\text{min}\right),$$

where 1.36 is the amount of oxygen bound per gram of haemoglobin at one atmosphere pressure (Hüfner’s constant). CDO_2_ measures were also divided into quintile ranks (Supplementary Table 1).

#### Neurodevelopmental assessment

Forty-six infants with CHD [42 born ≥37 weeks; Excluded participants: Died before 22 months = 4; declined = 8 (2 due to living overseas); postponed due to Covid-19 = 4; age <22 months = 4] attended a follow-up assessment at a median (IQR) of 22.2 (22.0–22.6) months (Table 1). Infants completed the *Bayley Scales of Infant and Toddler Development*–Third Edition (Bayley-III)^38^ administered by a developmental paediatrician (AC) to obtain cognitive and motor composite scores [test mean (SD) = 100(15)].

#### Socioeconomic status

Index of multiple deprivation (IMD) was calculated from postcode at birth for all infants who attended the follow-up assessment. IMD is a composite measure of socioeconomic status in England encompassing factors such as income, employment, education, health and crime (http://imd-by-postcode.opendatacommunities.org/; Accessed 02 June 2020). IMD was calculated from the 2015 data release and reported as scores and quintiles (most to least deprived). It was not possible to calculate IMD for one infant with CHD.

### Modelling typical development

#### Normative sample

A total of 219 healthy infants born ≥37 weeks [median(IQR) age at birth = 40.1 (39.1–41) gestational weeks; 109 male] recruited from prenatal or postnatal wards at St Thomas’ Hospital as part of the Developing Human Connectome Project were used to generate the normative model^24^ (dHCP; http://www.developingconnectome.org/; Accessed 03 February 2021). Exclusion criteria included admission to neonatal intensive care unit, major lesions identified on MRI such as perinatal arterial ischemic stroke or parenchymal haemorrhage and Bayley-III Cognitive or Motor Composite Scores below 70 (<2 SD from the test mean) at 18 months.

#### Magnetic resonance imaging

**Figure 1:**
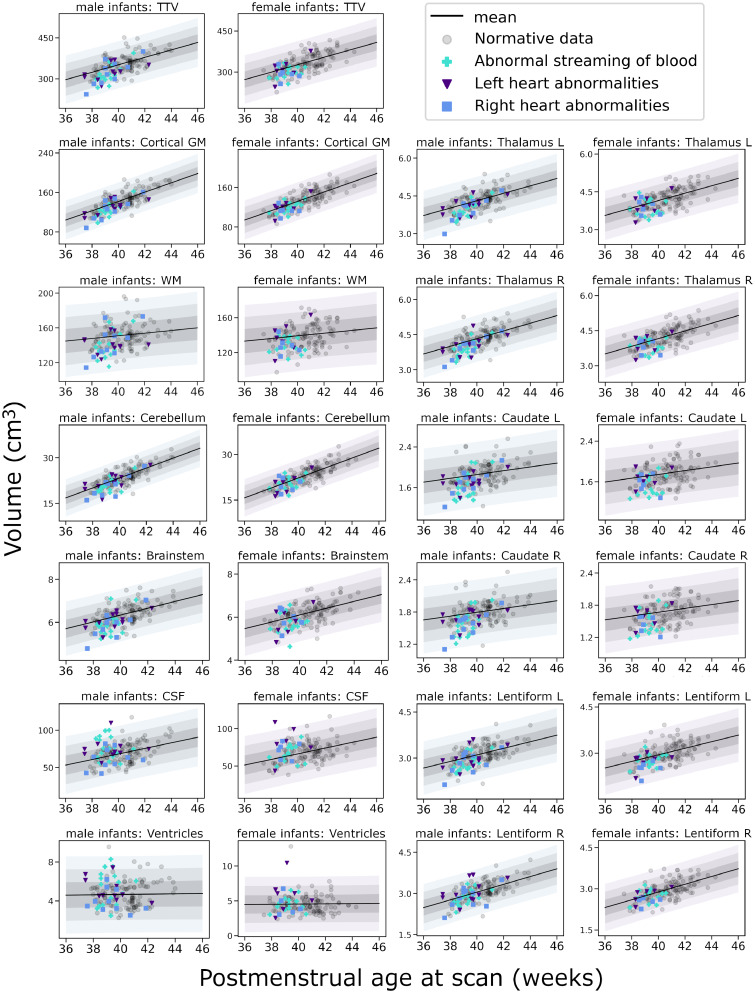
**Volumetric brain development in neonates with CHD.** Shaded areas represent ±1, 2 and 3 standard deviations from the normative model mean, separately for female and male infants. TTV = total tissue volume; GM = grey matter; WM = white matter; R = right; L = left.

All infants between 37 and 45 weeks postmenstrual age underwent MRI with the T1- and T2-weighted protocol as described previously [postmenstrual age at scan median (IQR) = 40.7 (39.4–41.4) weeks]. The normative sample was recruited and processed according to previously published methods^24^ and was used to model typical volumetric brain development (Fig. 1).

#### Gaussian process regression

Gaussian process regression (GPR), a Bayesian non-parametric regression method, implemented in GPy in Python (https://sheffieldml.github.io/GPy/; Accessed 16 December 2020) was used to model volumetric brain development from 37 to 45 weeks postmenstrual age.^24^ Briefly, GPR predicts individual data points and measures predictive confidence for each estimate. This confidence represents the distance between individual observations and the normative group mean at a point on the developmental trajectory.^21–23^ The difference between predicted and observed values normalized by the predictive confidence represents the deviation of a datapoint from the expected mean given the child’s postmenstrual age at scan, days of life and sex. This provides a z-score quantifying the degree of atypicality in each volume from each baby with CHD. GPR was estimated separately for total tissue volume, cortical grey matter, white matter, cerebellum, brainstem, extracerebral CSF, ventricles, left/right caudate, left/right lentiform and left/right thalamus.

#### Statistical analysis

Analyses were undertaken for the whole sample (*N* = 66) and separately for those infants born ≥37 weeks GA (*N* = 62). Extreme deviations from normative volumetric development were taken as an atypicality index of >±2.6 (corresponding to *P* < 0.005), representing the top and bottom 0.5% of the typical population.^22^ Fisher’s exact test was used to compare the proportion of deviations across the type of CHD (abnormal streaming of blood, left-sided heart lesions and right-sided heart lesions). Infants with extreme deviations were examined for cardiac diagnosis, gestational age at birth, birth weight and head circumference z-score, sex, cerebral oxygen delivery and brain injury.

Shapiro-Wilk was used to test normality. Kruskal-Wallis one-way test of variance was used to compare atypicality indices between the types of CHD. Partial Spearman’s Rank correlations were used to characterize the relationship between atypicality indices and Bayley-III scores including IMD as a covariate. The relationship between birth weight z-score and outcome scores was also tested to determine whether any relationship with atypicality indices reflected a multi-system impairment in development. Pearson correlations were used to test for associations between cerebral oxygen delivery and atypicality indices. Spearman’s Rank correlations were used to test the associations between IMD and outcome scores and atypicality indices. Multiple linear regression was used to predict outcome scores. Benjamini & Hochberg False Discovery Rate was applied to correct for multiple comparisons (reported as p_FDR_). All analyses were performed in R v3.6.2.

Causal mediation analysis can be used to characterize aetiological mechanisms underlying relationships between independent and dependent variables through a third ‘mediating’ variable.^39^ In this study, causal mediation analysis was used to test whether lower cerebral oxygen delivery in the neonatal period was associated with poor early neurodevelopmental abilities through an effect on volumetric atypicality indices. Causal mediation analysis was used to test for a mediation effect when significant associations between atypicality indices and both outcome scores and cerebral oxygen delivery were identified. The analysis was conducted using the Mediation package in R v3.6.2.^40^ Linear regression models were used to predict (a) outcome scores from cerebral oxygen delivery and atypicality index with IMD as covariate; (b) outcome scores from atypicality index with IMD as a covariate and (c) atypicality index from cerebral oxygen delivery. Given the theoretical foundations for this analysis, our primary aim was to test for a significant indirect effect of cerebral oxygen delivery on cognitive outcome through the mediating effect of atypicality indices.^41^ The indirect effect was computed across 1000 bootstrapped samples to calculate the average causal mediation effect (ACME) with quasi-Bayesian 95% confidence intervals.

Causal mediation analysis assumes no unmeasured confounding variables affecting both mediator and outcome and therefore no shared unexplained variance in regression models (a) and (c) described above. In observational studies, sensitivity analysis is used to quantify the degree to which this assumption must be violated to alter the direction of the effect and therefore the study outcome.^41^ The method used here uses a correlation parameter (*ρ*) to test for omitted confounding variables which regress onto both the mediator and outcome.^39^^,^^40^ A simulated confounding variable was introduced into the calculation of the ACME across 1000 simulations. The influence of this variable on the model was incrementally increased to determine the ‘critical *ρ*’ at which the indirect effect reverses direction, therefore altering the study conclusions. A large ‘critical *ρ*’ suggests a potential unobserved confounding variable must be highly correlated with both mediator and outcome to alter the result and therefore that the result is robust to unmeasured variables.^39^

### Data availability

The data that support the findings of this study are available from the corresponding author upon reasonable request.

## Results

The primary cardiac diagnoses and demographic characteristics of infants with CHD are summarized in Table 1. Of the infants who underwent a septostomy (*N* = 17), two were scanned before the procedure. Four infants in the sample were born at 34–37 weeks gestation (tricuspid atresia 34 + 6; pulmonary atresia 35 + 2; pulmonary atresia 35 + 5; TGA 36 + 3). Demographic information for infants with CHD born ≥37 weeks are also summarized in Table 1.

### Volumetric atypicality Indices in infants with CHD

Mean atypicality indices for all tissue volumes in infants with CHD were half a standard deviation below the predicted group mean except the right lentiform nucleus (Fig. 1; Table 2). In addition, infants with CHD had larger extracerebral CSF spaces than the predicted normative mean. By contrast, the median atypicality index of ventricle volume was in-line with the normative model. The results were not different when examining infants born ≥37 weeks (Supplementary Table 2). There were no significant differences in atypicality indices between subgroups of CHD across the whole sample (Table 2; p_FDR_ ≥0.24 in all analyses) or in infants born ≥37 weeks (Supplementary Table 2; p_FDR_ ≥0.31 in all analyses).

**Table 2:** Atypicality indices in infants with CHD

Region	Whole group	Abnormal streaming of blood	Left heart abnormalities	Right heart abnormalities	Kruskal wallis H (pFDR)	Extreme negative deviations	Extreme positive deviations
	
	Atypicality index mean (SD)	Atypicality index median (IQR)	Atypicality index median (IQR)	Atypicality index median (IQR)		Number (%)	Number (%)
Cortical grey matter	−0.58 (0.88)	−0.63 (−1.04 to −0.21)	−0.56 (−0.84–0.31)	−0.75 (−1.46 to −0.21)	1.7 (0.47)	0 (0)	0 (0)
White matter	−0.59 (0.99)	−0.88 (−1.24 to −0.16)	−0.51 (−0.99–0.40)	−0.79 (−1.29 to −0.04)	1.7 (0.47)	0 (0)	0 (0)
Extracerebral CSF	0.57 (1.21)	0.40 (0.02–1.00)	0.79 (0.14–1.59)	−0.01 (−0.90–1.02)	3.9 (0.33)	0 (0)	5 (8)
Ventricles median (IQR)	0.01 (−0.76–1.16)	0.08 (0.79–1.16)	0.22 (−0.12–1.20)	−0.53 (−1.06–0.35)	5.2 (0.23)	0 (0)	2 (3)
Cerebellum	−0.43 (1.02)	−0.41 (−0.89–0.04)	0.19 (−1.05 –0.63)	−0.57 (−1.63 to −0.09)	3.0 (0.36)	1 (2)	0 (0)
Brainstem	−0.60 (1.13)	−0.90 (−1.37–0.40)	−0.27 (−1.07 –0.40)	−0.77 (−1.17–0.11)	2.3 (0.46)	2 (3)	0 (0)
Left thalamus	−0.57 (0.97)	−0.85 (−1.45 to −0.27)	−0.26 (−0.52–0.56)	−0.26 (−1.39–0.02)	3.8 (0.33)	1 (2)	0 (0)
Right thalamus	−0.66 (0.93)	−0.96 (−1.40 to −0.14)	−0.43 (−0.94–0.23)	−0.40 (−1.44 to −0.09)	1.4 (0.50)	0 (0)	0 (0)
Left lentiform	−0.52 (0.89)	−0.58 (−1.13 to −0.22)	−0.39 (−0.68–0.29)	−0.79 (−1.47 to −0.03)	3.4 (0.34)	0 (0)	0 (0)
Right lentiform	−0.16 (0.97)	−0.27 (−0.87–0.19)	0.40 (−0.34–0.71)	−0.20 (−1.16–0.06)	5.7 (0.24)	0 (0)	0 (0)
Left caudate nucleus	−0.71 (0.96)	−1.27 (−1.78 to −0.23)	−0.34 (−0.71–0.22)	−0.43 (−1.31 to −0.03)	5.8 (0.24)	1 (2)	0 (0)
Right caudate nucleus	−0.76 (1.00)	−1.12 (−1.82 to −0.16)	−0.17 (−0.75–0.32)	−0.69 (−1.72–0.36)	6.6 (0.24)	1 (1.52)	0 (0)
Total tissue volume	−0.62 (0.94)	−0.87 (−1.12 to −0.31)	−0.47 (−0.96–0.38)	−0.72 (−1.55 to −0.17)	1.7 (0.47)	1 (1.52)	0 (0)

### Extreme deviations from the normative model mean

The frequency of extreme deviations in each regional volume is summarized in Table 2. The most common extreme deviation was an enlargement of the extracerebral CSF occurring in 8% of babies with CHD. Extreme positive deviations (atypicality index >2.6) indicating extremely high volume for a given postmenstrual age, days of life and sex, were also identified in the ventricles. Extreme negative deviations (atypicality index <−2.6), indicating extremely low volume for a given postmenstrual age, days of life and sex, were identified in the brainstem, cerebellum, bilateral caudate nuclei, left thalamus and total tissue volume. There were no extreme deviations in cortical grey matter, white matter, right thalamus and lentiform nuclei volumes.

Nine infants (14%) showed an extreme deviation in at least one regional volume: 3 TGA, 3 CoA, 1 HLHS and 2 tricuspid atresia (See supplementary Table 3 for clinical information). The largest number of deviations reported in one infant was 5. This was a male infant with tricuspid atresia and additional abnormalities born at 34 + 6 weeks, scanned at a postmenstrual age of 37 + 4 weeks, who had severe WMI. All other infants with extreme deviations were born after 38 weeks gestation. All infants with extreme deviations in brain development were diagnosed with CHD antenatally and were inborn.

The largest proportion of infants with extreme deviations was seen in the group with left-sided cardiac lesions (22%) followed by abnormalities in the right side of the heart (13%; 8% in infants born ≥37 weeks) with the fewest seen in abnormal streaming of blood (9%; 9% in infants born ≥37 weeks); however, this was not statistically significant [abnormal streaming vs left-sided, odds ratio (OR) (95% CI) = 0.37 (0.05–2.51), p_FDR_ = 0.99; Abnormal streaming vs right-sided, OR (95% CI) = 0.73 (0.07–101.74) p_FDR_ = 1.00; left-sided vs right-sided, OR (95% CI) = 1.96 (0.24–25.03) p_FDR_ = 0.70)]. There were no significant differences in the proportion of extreme deviations between subgroups of CHD when analysing infants born ≥37 weeks (p_FDR_ ≥0.69 in all analyses).

### Neurodevelopmental abilities at 22 months in infants with CHD

Cognitive and motor composite scores and socioeconomic status in children with CHD who attended the follow-up assessment (*N* = 46; born ≥37 weeks *N* = 42) are summarized in Table 3.

**Table 3:** Demographics at follow-up for infants with CHD

	All infants (*N* = 46)	Infants born ≥37 weeks (*N* = 42)
Age at follow-up corrected for gestational age at birth median (IQR)	22.2 (22.0–22.6)	22.2 (22.0–23.2)
Cognitive composite score mean (SD)	93 (10)	93 (10)
Motor composite score mean (SD)	94 (10)	95 (10)
Index of multiple deprivation score median (IQR)	21.9 (13.4–30.8)	21.9 (13.1–30.8)
Index of multiple deprivation quintiles	Number (%)	
First (most deprived)	10 (22)	9 (22)
Second	13 (29)	12 (29)
Third	8 (18)	6 (15)
Fourth	9 (20)	9 (22)
Fifth	5 (11)	5 (12)

### Associations between volumetric atypicality indices and outcome

Cognitive composite scores were positively correlated with atypicality index in the bilateral thalamus and caudate and left lentiform nucleus after correcting for IMD across the whole sample (Table 4). In children with CHD born ≥37 weeks, the cognitive outcome was significantly associated with these subcortical structures and also with cortical grey matter and total tissue atypicality indices. Motor composite scores were not correlated with any atypicality indices. Birth weight z-score (whole sample: cognitive: *ρ* = 0.20 p_FDR_ = 0.37, motor: *ρ* = 0.08 p_FDR_ = 0.61; born ≥37 weeks: cognitive: *ρ* = 0.29 p_FDR_ = 0.14, motor: *ρ* = 0.06 p_FDR_ = 0.72) and IMD (whole sample: cognitive: *ρ*=−0.24, p_FDR_ = 0.21, motor: *ρ* = 0, p_FDR_ = 0.10; born ≥37 weeks: cognitive: *ρ*=−0.26 p_FDR_ = 0.19, Motor: *ρ*=−0.05 p_FDR_ = 0.74) were not associated with Bayley-III scores. IMD was not significantly associated with atypicality indices (Supplementary Table 4; *ρ* ≤ 0.35 p_FDR_≥0.23 in all analyses).

**Table 4:** Relationship between Bayley-III scores and brain volume atypicality indices

Region	Cognitive composite score		Motor composite score	
	Whole sample Spearman’s *ρ* (pFDR)	Infants born ≥37 weeks Spearman’s *ρ* (pFDR)	Whole sample Spearman’s *ρ* (pFDR)	Infants born ≥37 weeks Spearman’s *ρ* (pFDR)
Extracerebral CSF	−0.07 (1.00)	−0.02 (0.87)	0.004 (0.98)	−0.08 (0.67)
Cortical grey matter	0.31 (0.09)	**0.40 (0.03)**	0.07 (0.72)	0.07 (0.67)
White matter	0.25 (0.16)	0.31 (0.08)	0.12 (0.62)	0.13 (0.62)
Ventricles	0.13 (0.46)	0.14 (0.42)	−0.10(0.62)	−0.15 (0.62)
Cerebellum	0.24 (0.39)	0.24 (0.17)	0.10 (0.62)	0.09 (0.67)
Brainstem	0.11 (0.72)	0.16 (0.39)	0.12 (0.62)	0.11 (0.67)
Left thalamus	**0.39 (0.04)**	**0.45 (0.02)**	0.31 (0.28)	0.24 (0.32)
Right thalamus	**0.37 (0.04)**	**0.44 (0.02)**	0.23 (0.37)	0.24 (0.32)
Left caudate	**0.38 (0.04)**	**0.44 (0.02)**	0.31 (0.28)	0.28 (0.31)
Right caudate	**0.37 (0.04)**	**0.46 (0.02)**	0.31 (0.28)	0.29 (0.31)
Left lentiform	**0.36 (0.04)**	**0.42 (0.02)**	0.21 (0.37)	0.27 (0.31)
Right lentiform	0.26(0.15)	0.32 (0.08)	0.25 (0.37)	0.29 (0.31)
Total tissue volume	0.30 (0.10)	**0.40 (0.03)**	0.13 (0.62)	0.15 (0.62)

Results in bold are significant.

**Figure 2:**
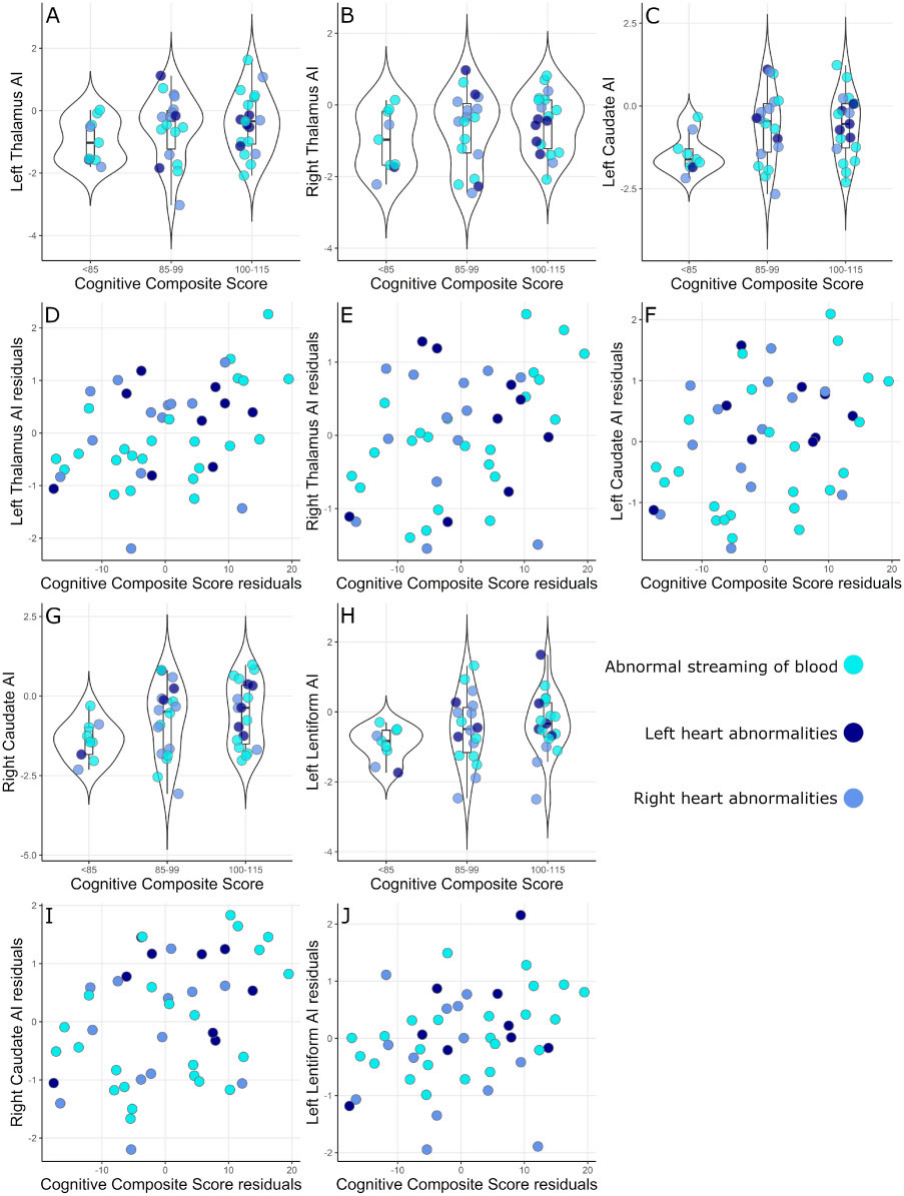
**Associations between cognitive scores and atypicality indices.** Box plots showing the relationship between cognitive composite score and (**A**) left thalamus, (**B**) right thalamus, (**C**) left caudate, (**G**) right caudate, (**H**) and left lentiform atypicality indices across the whole sample. Scatter plots showing cognitive composite score residuals plotted against (**D**) left thalamus, (**E**) right thalamus, (**F**) left caudate, (**I**) right caudate and (**J**) left lentiform AI residuals across the whole sample. Residuals are corrected for the index of multiple deprivations.

Linear regression revealed atypicality indices in bilateral thalami and caudate nuclei and left lentiform nucleus, together with IMD, explained 17–23% of the variance in cognitive composite scores across the whole sample (Table 5; Fig. 2). In infants born ≥37 weeks bilateral thalami and caudate nuclei, left lentiform nuclei, total tissue volume and cortical grey matter atypicality indices, together with IMD, explained 19–33% of the variance in cognitive composite scores (supplementary Table 5; Supplementary Fig. 1).

**Table 5:** Regression coefficients for models predicting cognitive composite score

Variable	Β	*P*
Cognitive composite score ∼ left thalamus atypicality index + index of multiple deprivation		
Left thalamus atypicality index	0.41	0.006
Index of multiple deprivation	−0.42	0.005
*R^2^ [95% CI] = 0.23 [0.02–0.47]; adjusted R^2^ = 0.19; F(2,42) = 6.25, p_FDR_ = 0.01*		
Cognitive composite score ∼ right thalamus atypicality index + index of multiple deprivation		
Right thalamus atypicality index	0.39	0.011
Index of multiple deprivation	−0.41	0.007
*R^2^ [95% CI] = 0.21 [0.02–0.43]; adjusted R^2^ = 0.17; F(2,42) = 5.64, p_FDR_ = 0.01*		
Cognitive composite score ∼ left caudate nucleus atypicality index + index of multiple deprivation		
Left caudate nucleus atypicality index	0.33	0.017
Index of multiple deprivation	−0.35	0.019
*R^2^ [95% CI] = 0.20 [0.01–0.37]; adjusted R^2^ = 0.16; F(2,42) = 5.12, p_FDR_ = 0.01*		
Cognitive composite score ∼ right caudate nucleus atypicality index + index of multiple deprivation		
Right caudate nucleus atypicality index	0.35	0.016
Index of multiple deprivation	−0.31	0.030
*R^2^ [95% CI] = 0.20 [0.03–0.39]; adjusted R^2^ = 0.16; F(2,42) = 5.20, p_FDR_ = 0.01*		
Cognitive composite score ∼ left lentiform nucleus atypicality index + index of multiple deprivation		
Left lentiform nucleus atypicality index	0.30	0.043	
Index of multiple deprivation	−0.27	0.064	
*R^2^ [95% CI] = 0.17 [0.01–0.39]; adjusted R^2^ = 0.13; F(2,42) = 4.16, p_FDR_ = 0.02*		

### Associations between CDO_2_, brain volumes and cognitive outcome

Across the whole sample, CDO_2_ was positively correlated with all brain tissue volume atypicality indices (supplementary Table 6; p_FDR_ ≤0.047 and *r* ≥ 0.29 in all analyses) but not with extracerebral CSF (*r* = 0.11 p_FDR_ = 0.48) or ventricle (*r* = 0.08 p_FDR_ = 0.56) atypicality indices. In infants with CHD born ≥37 weeks, CDO_2_ was positively correlated with total tissue volume, left thalamus, cortical grey matter, brainstem and bilateral lentiform nuclei atypicality indices (Supplementary Table 6 p_FDR_ ≤0.049 and *r* ≥ 0.32 in all analyses). CDO_2_ did not correlate with cognitive (whole sample: *ρ* = 0.16 *P* = 0.34; infants born ≥37 weeks *ρ* = 0.17 *P* = 0.32) or motor (whole sample: *ρ* = 0.14 *P* = 0.42; infants born ≥37 weeks *ρ* = 0.10 *P* = 0.57) composite scores when co-varying for IMD.

**Figure 3:**
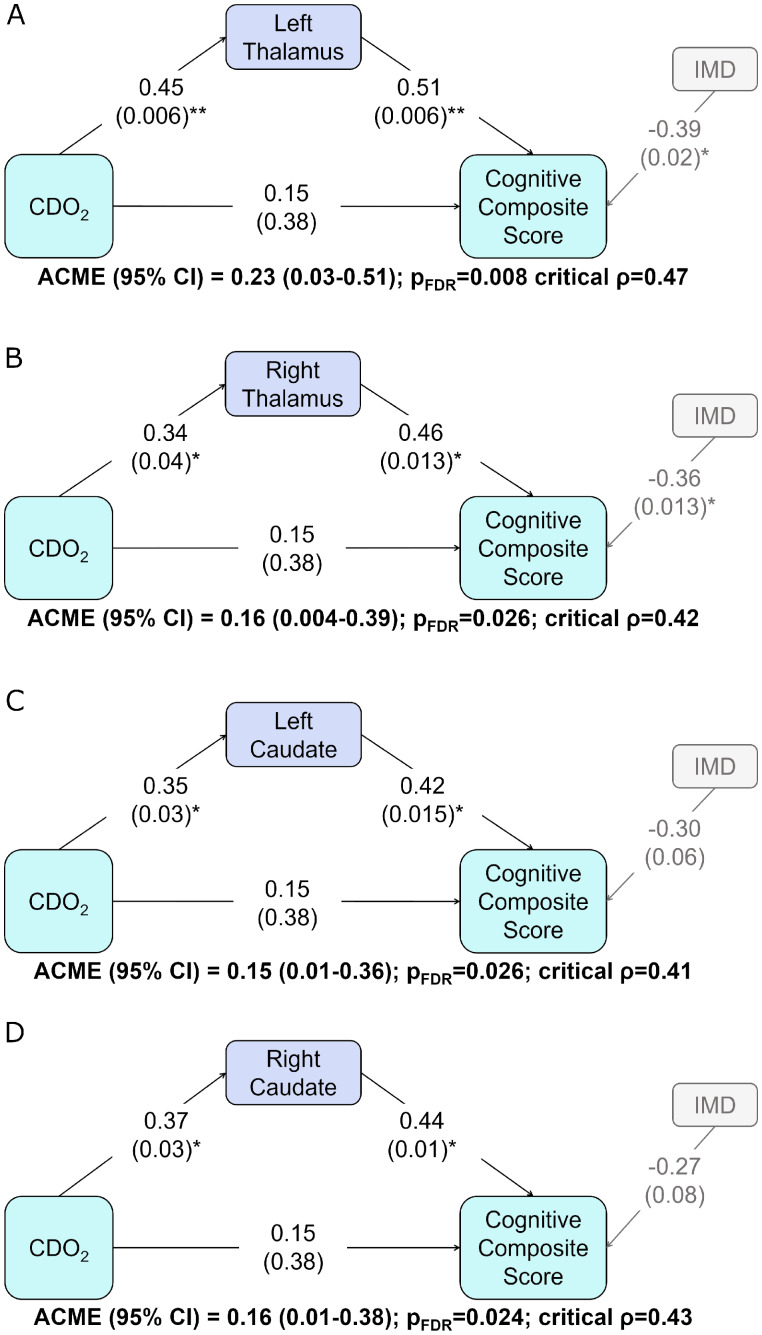
**The relationship between CDO_2_ and cognitive composite scores mediated by atypicality indices.** Path diagrams showing the indirect relationship between CDO_2_ and cognitive composite score mediated by (**A**) left thalamus, (**B**) right thalamus, (**C**) left caudate nucleus and (**D**) right caudate nucleus atypicality indices across the whole sample. Standardized regression coefficients are reported; numbers in brackets show *P*-value. *P < 0.05; **P < 0.01. ACME = average causal mediation effect; CDO_2_ = cerebral oxygen delivery.

Across the whole sample, reduced CDO_2_ was indirectly associated with lower cognitive composite scores through the mediating effect of atypicality indices in the thalami and caudate nuclei (Fig. 3). Sensitivity analysis revealed the *ρ* at which the direction of the indirect effect reversed was 0.41–0.47 for all analyses, suggesting a robust effect. There was no significant mediation effect of left lentiform nucleus atypicality index on the relationship between CDO_2_ and cognitive outcome [ACME (95% CI) = 0.17 (−0.05–0.44) p_FDR_ = 0.15]. In infants with CHD born ≥37 weeks, reduced CDO_2_ was indirectly associated with lower cognitive composite scores through the mediating effect of atypicality indices in the left thalamus, left lentiform nucleus, cortical grey matter and total tissue volume (Supplementary Fig. 2). Sensitivity analysis revealed the *ρ* at which the direction of the indirect effect reversed was 0.40–0.56 for all analyses, suggesting a robust effect.

## Discussion

This study applied normative modelling techniques to characterize volumetric brain development in neonates with CHD before surgery and to assess the relationship between atypical brain development and subsequent neurodevelopmental outcome in early childhood. Lower than predicted volumes for a given postmenstrual age, days of life and sex in the deep grey matter were associated with poorer cognitive abilities at 22 months. In those infants born ≥37 weeks, poor cognitive abilities were also associated with negative cortical grey matter and total tissue volume atypicality indices. Reduced CDO_2_ was indirectly associated with poor cognitive outcome in early childhood through the mediating effect of reduced volumetric brain development in these regions. Extreme deviations from typical brain development were reported in over 13% of infants with the most common being increased extracerebral CSF volume as well as increased ventricle and decreased subcortical grey matter volumes.

Consistent with previous studies,^11^^,^^15^ including in this cohort,^12^ we observed that infants with CHD had reduced brain tissue volumes and increased extracerebral CSF before surgery. However, to our knowledge, this is the first use of a normative modelling approach to assess brain development in individual infants with CHD. Our results highlight the heterogeneity of intracranial development in CHD, with atypicality indices ranging from extremely abnormal to close to the typical mean for a given postmenstrual age, days of life and sex.

Fetal brain development accelerates in the third trimester of pregnancy and is accompanied by an increase in cerebral blood flow.^42^^,^^43^ This is characterized by the dilation of cerebral arteries, measured as a decrease in pulsatility index on fetal doppler ultrasound, to meet increased metabolic demands in the fetal brain.^44^ Pulsatility index is further reduced in infants with CHD to increase cerebral blood flow to compensate for reduced cardiac output or substrate delivery.^45^^,^^46^ Foetuses with left-sided lesions have reduced cerebral perfusion and impaired cerebral substrate delivery whereas foetuses with TGA show intact cerebral blood flow but reduced cerebral substrate delivery.^43^^,^^46^^,^^47^ By contrast, the pulsatility index in the middle cerebral artery is the same or higher in foetuses with right-sided lesions compared with controls.^48–50^ Infants with left-sided cardiac lesions may be at particular risk of extreme deviations in brain development due to reduced cerebral blood flow and substrate delivery *inutero*. In our analysis, the largest proportion of extreme deviations in brain volumes were identified in infants with left-sided cardiac lesions; however, the differences between groups were not significant.

The thalamus and caudate have been implicated in cognitive abilities in children born prematurely; subcortical volumetric development is altered and has been related to later cognitive abilities^51^; higher thalamocortical connectivity at term equivalent age is associated with improved cognitive abilities at 2 years^52^ and improved postnatal caudate growth has been associated with higher full-scale IQ at 4 years,^53^ suggesting that the development of the thalamus and the caudate nucleus is important for cognitive abilities across childhood. It has previously been hypothesized that subcortical structures are potential mediators of poor neurodevelopment in infants with CHD.^54^ Lower basal ganglia and thalamus volumes postoperatively have been associated with lower IQ scores at 6 years.^20^ CDO_2_ and cerebral blood flow are lower in infants with cyanotic compared with acyanotic CHD^55^ and poor behavioural state regulation in neonates with cyanotic CHD, but not acyanotic CHD, has also been linked with lower total subcortical grey matter volume before surgery.^56^ We provide evidence that lower CDO_2_ resulting in impaired deep grey matter development may lie on the aetiological pathway linking CHD to poor early cognitive abilities.

In those infants with CHD born ≥37 weeks, impaired cortical grey matter and total tissue volumetric development were also implicated in this pathway. We have previously demonstrated that grey matter volume, gyrification and cortical microstructural development are impaired in this cohort relative to controls and that the degree of cortical developmental impairment was associated with reduced CDO_2_.^14^^,^^15^ In addition, previous research has identified associations between reduced CDO_2_ and lower total brain volume in foetuses with CHD.^13^ A small study demonstrated a relationship between increased foetal brain volume and higher average Bayley-III scores at 6 months.^45^ Furthermore, higher postsurgical, but not presurgical total brain volume has been linked with higher cognitive scores at 1 year^19^ and larger total brain volumes at 9 years are associated with higher full-scale IQ in children with CHD.^57^

### Study limitations

This study included a heterogeneous group of cardiac diagnoses and our sample size of CHD infants with neurodevelopmental outcome was not large. We focused on the early outcome and further studies with longitudinal analysis are required to determine whether neonatal brain volumes relate to IQ or academic attainment in childhood and beyond. In addition, CDO_2_ was measured at a single timepoint *exutero* and this measure may not capture fully the disruption in cerebral oxygenation experienced by foetuses and infants with CHD.

## Conclusions

Infants with CHD are at increased risk of extreme deviations from typical neonatal brain volumetric development before surgery, particularly involving reduced subcortical grey matter volumes and expansion of extracerebral CSF and ventricle volumes. Negative deviations in deep grey matter, cortical grey matter and total tissue volume in the neonatal period were associated with poorer cognitive abilities at 22 months. We provide the first evidence that the aetiology of poor early cognitive abilities may encompass reduced CDO_2_ associated with impaired grey matter development. Further research to identify interventions that promote both CDO_2_ and grey matter growth may improve early cognitive outcomes in infants with CHD.

## Supplementary material

Supplementary material is available at *Brain Communications* online.

## Supplementary Material

fcab046_Supplementary_Data

## Abbreviations

ACME =: average causal mediation effect
AI =: atypicality index
Bayley-III =: *Bayley Scales of Infant and Toddler Development*–Third edition
CDO2 =: cerebral oxygen delivery
CHD =: congenital heart disease
CoA =: coarctation of the aorta
dHCP =: Developing Human Connectome Project
GM =: grey matter
GPR =: Gaussian process regression
HLHS =: hypoplastic left heart syndrome
IMD =: Index of Multiple Deprivation
IQR =: interquartile range
L =: left
OR =: odds ratio
R =: right
TE =: echo time
TGA =: dextro-transposition of the great arteries
TI =: inversion time
TR =: repetition time
TTV =: total tissue volume
WM =: white matter
WMI =: white matter injury

## References

[1] EUROCAT. Eurocat Prevalence Tables. 2015. http://www.eurocat-network.eu/accessprevalencedata/prevalencetables. Accessed 06 November 2019.

[2] Wren C , Sullivan JJ. Survival with congenital heart disease and need for follow up in adult life. Heart. 2001;85(4):438–443. 11250973 10.1136/heart.85.4.438 PMC1729699

[3] Latal B. Neurodevelopmental outcomes of the child with congenital heart disease. Clin Perinatol. 2016;43(1):173–185. 26876129 10.1016/j.clp.2015.11.012

[4] Marino BS , Lipkin PH, Newburger JW, et al Neurodevelopmental outcomes in children with congenital heart disease: Evaluation and management: A scientific statement from the American Heart Association. Circulation. 2012;126(9):1143–1172. 22851541 10.1161/CIR.0b013e318265ee8a

[5] Bellinger DC , Wypij D, Rivkin MJ, et al Adolescents with d-transposition of the great arteries corrected with the arterial switch procedure: Neuropsychological assessment and structural brain imaging. Circulation. 2011;124(12):1361–1369. 21875911 10.1161/CIRCULATIONAHA.111.026963 PMC3217719

[6] Klouda L , Franklin WJ, Saraf A, Parekh DR, Schwartz DD. Neurocognitive and executive functioning in adult survivors of congenital heart disease. Congenit Heart Dis. 2017;12(1):91–98. 27650247 10.1111/chd.12409

[7] Dewey D , Volkovinskaia A. Health-related quality of life and peer relationships in adolescents with developmental coordination disorder and attention-deficit-hyperactivity disorder. Dev Med Child Neurol. 2018;60(7):711–717. 29611868 10.1111/dmcn.13753

[8] Lawley CM , Winlaw DS, Sholler GF, et al School-age developmental and educational outcomes following cardiac procedures in the first year of life: A Population-Based Record Linkage Study. Pediatr Cardiol. 2019;40(3):570–579. 30535960 10.1007/s00246-018-2029-y

[9] Bouyssi-Kobar M , Limperopoulos C, Clouchoux C, et al Delayed cortical development in fetuses with complex congenital heart disease. Cereb Cortex. 2013;23(12):2932–2943. 22977063 10.1093/cercor/bhs281

[10] Limperopoulos C , Tworetzky W, McElhinney DB, et al Brain volume and metabolism in fetuses with congenital heart disease: Evaluation with quantitative magnetic resonance imaging and spectroscopy. Circulation. 2010;121(1):26–33. 20026783 10.1161/CIRCULATIONAHA.109.865568 PMC2819908

[11] von Rhein M , Buchmann A, Hagmann C, et al Severe congenital heart defects are associated with global reduction of neonatal brain volumes. J Pediatr. 2015;167(6):1259–1263.e1. 26233604 10.1016/j.jpeds.2015.07.006

[12] Ng IHX , Bonthrone AF, Kelly CJ, et al Investigating altered brain development in infants with congenital heart disease using tensor-based morphometry. Sci Rep. 2020;10(1):14909. 32913193 10.1038/s41598-020-72009-3 PMC7483731

[13] Sun L , Macgowan CK, Sled JG, et al Reduced fetal cerebral oxygen consumption is associated with smaller brain size in fetuses with congenital heart disease. Circulation. 2015;131(15):1313–1323. 25762062 10.1161/CIRCULATIONAHA.114.013051 PMC4398654

[14] Kelly CJ , Christiaens D, Batalle D, et al Abnormal microstructural development of the cerebral cortex in neonates with congenital heart disease is associated with impaired cerebral oxygen delivery. J Am Heart Assoc. 2019;8(5):e009893. 30821171 10.1161/JAHA.118.009893 PMC6474935

[15] Kelly CJ , Makropoulos A, Cordero-Grande L, et al Impaired development of the cerebral cortex in infants with congenital heart disease is correlated to reduced cerebral oxygen delivery. Sci Rep. 2017;7(1):15088. 29118365 10.1038/s41598-017-14939-z PMC5678433

[16] Claessens NHP , Kelly CJ, Counsell SJ, Benders MJNL. Neuroimaging, cardiovascular physiology, and functional outcomes in infants with congenital heart disease. Dev Med Child Neurol. 2017;59(9):894–902. 28542743 10.1111/dmcn.13461

[17] Knirsch W , Mayer KN, Scheer I, et al Structural cerebral abnormalities and neurodevelopmental status in single ventricle congenital heart disease before Fontan procedure. Eur J Cardiothorac Surg. 2017;51(4):740–746. 28013288 10.1093/ejcts/ezw399

[18] Heye KN , Knirsch W, Latal B, et al Reduction of brain volumes after neonatal cardiopulmonary bypass surgery in single-ventricle congenital heart disease before Fontan completion. Pediatr Res. 2018;83(1-1):63–70. 29278641 10.1038/pr.2017.203

[19] Meuwly E , Feldmann M, Knirsch W, et al Postoperative brain volumes are associated with one-year neurodevelopmental outcome in children with severe congenital heart disease. Sci Rep. 2019;9(1):10885. 31350426 10.1038/s41598-019-47328-9 PMC6659678

[20] Claessens NHP , Algra SO, Ouwehand TL, et al Perioperative neonatal brain injury is associated with worse school-age neurodevelopment in children with critical congenital heart disease. Dev Med Child Neurol. 2018;60(10):1052–1058. 29572821 10.1111/dmcn.13747

[21] O'Muircheartaigh J , Robinson EC, Pietsch M, et al Modelling brain development to detect white matter injury in term and preterm born neonates. Brain. 2020;143(2):467–479. 31942938 10.1093/brain/awz412 PMC7009541

[22] Dimitrova R , Pietsch M, Christiaens D, et al Heterogeneity in brain microstructural development following preterm birth. Cereb Cortex. 2020;30(9):4800–4810. 32306044 10.1093/cercor/bhaa069 PMC7391275

[23] Marquand AF , Rezek I, Buitelaar J, Beckmann CF. Understanding heterogeneity in clinical cohorts using normative models: Beyond case-control studies. Biol Psychiatry. 2016;80(7):552–561. 26927419 10.1016/j.biopsych.2015.12.023 PMC5023321

[24] Dimitrova R , Arulkumaran S, Carney O, et al Phenotyping the preterm brain: Characterising individual deviations from normative volumetric development in two large infant cohorts. [published online January 1, 2020]. *bioRxiv*. doi:10.1101/2020.08.05.228700 10.1093/cercor/bhab039 PMC8258435 33822913

[25] Ewer AK , Middleton LJ, Furmston AT, et al Pulse oximetry screening for congenital heart defects in newborn infants (PulseOx): A test accuracy study. Lancet. 2011;378(9793):785–794. 21820732 10.1016/S0140-6736(11)60753-8

[26] Cole TJ , Freeman JV, Preece MA. British 1990 growth reference centiles for weight, height, body mass index and head circumference fitted by maximum penalized likelihood. Stat Med. 1998;17(4):407–429. 9496720

[27] Anderson RH , Becker AE, Freedom RM, et al Sequential segmental analysis of congenital heart disease. Pediatr Cardiol. 1984;5(4):281–287. 6533609 10.1007/BF02424973

[28] Hughes EJ , Winchman T, Padormo F, et al A dedicated neonatal brain imaging system. Magn Reson Med. 2017;78(2):794–804. 27643791 10.1002/mrm.26462 PMC5516134

[29] Cordero-Grande L , Hughes EJ, Hutter J, Price AN, Hajnal JV. Three-dimensional motion corrected sensitivity encoding reconstruction for multi-shot multi-slice MRI: Application to neonatal brain imaging. Magn Reson Med. 2018;79(3):1365–1376. 28626962 10.1002/mrm.26796 PMC5811842

[30] Cordero-Grande L , Teixeira RPAG, Hughes EJ, Hutter J, Price AN, Hajnal JV. Sensitivity encoding for aligned multishot magnetic resonance reconstruction. IEEE Trans Comput Imaging. 2016;2(3):266–280.

[31] Varela M , Groves AM, Arichi T, Hajnal JV. Mean cerebral blood flow measurements using phase contrast MRI in the first year of life. NMR Biomed. 2012;25(9):1063–1072. 22290659 10.1002/nbm.2771

[32] Kelly CJ , Arulkumaran S, Tristão Pereira C, et al Neuroimaging findings in newborns with congenital heart disease prior to surgery: An observational study. Arch Dis Child. 2019;104(11):1042–1048. 31243012 10.1136/archdischild-2018-314822 PMC6801127

[33] Beca J , Gunn JK, Coleman L, et al New white matter brain injury after infant heart surgery is associated with diagnostic group and the use of circulatory arrest. Circulation. 2013;127(9):971–979. 23371931 10.1161/CIRCULATIONAHA.112.001089

[34] Makropoulos A , Robinson EC, Schuh A, et al The developing human connectome project: A minimal processing pipeline for neonatal cortical surface reconstruction. Neuroimage. 2018;173:88–112. 29409960 10.1101/125526 PMC6783314

[35] Makropoulos A , Aljabar P, Wright R, et al Regional growth and atlasing of the developing human brain. Neuroimage. 2016;125:456–478. 26499811 10.1016/j.neuroimage.2015.10.047 PMC4692521

[36] Heiberg E , Sjögren J, Ugander M, Carlsson M, Engblom H, Arheden H. Design and validation of segment—Freely available software for cardiovascular image analysis. BMC Med Imaging. 2010;10:1. 20064248 10.1186/1471-2342-10-1 PMC2822815

[37] McLellan SA , Walsh TS. Oxygen delivery and haemoglobin. Contin Educ Anaesth Crit Care Pain. 2004;4(4):123–126.

[38] Bayley N. Bayley scales of infant and toddler development. 3rd ed. PsychCorp, San Antonio Texas, USA: Pearson; 2006.

[39] Zhang Z , Zheng C, Kim C, Van Poucke S, Lin S, Lan P. Causal mediation analysis in the context of clinical research. Ann Transl Med. 2016;4(21):425. 27942516 10.21037/atm.2016.11.11 PMC5124624

[40] Tingley D , Yamamoto T, Hirose K, Keele L, Imai K. Mediation: R package for causal mediation analysis [published online 2014].

[41] Agler R , De Boeck P. On the interpretation and use of mediation: Multiple perspectives on mediation analysis. Front Psychol. 2017;8:1984. https://www.frontiersin.org/article/10.3389/fpsyg.2017.01984. Accessed 04 June 2020. 29187828 10.3389/fpsyg.2017.01984 PMC5694788

[42] Du Plessis AJ. Cerebral blood flow and metabolism in the developing fetus. Clin Perinatol. 2009;36(3):531–548. 19732612 10.1016/j.clp.2009.07.002 PMC13093913

[43] Lee F-T , Seed M, Sun L, Marini D. Fetal brain issues in congenital heart disease. [published online 2020]. *Transl Pediatr*. http://tp.amegroups.com/article/view/51940. Accessed 02 February 2021. 10.21037/tp-20-224 PMC8429876 34584890

[44] Arduini D , Rizzo G. Normal values of Pulsatility Index from fetal vessels: A cross-sectional study on 1556 healthy fetuses. J Perinat Med. 1990;18(3):165–172. 2200862 10.1515/jpme.1990.18.3.165

[45] Masoller N , Sanz-Cortés M, Crispi F, et al Mid-gestation brain doppler and head biometry in fetuses with congenital heart disease predict abnormal brain development at birth. Ultrasound Obstet Gynecol. 2016;47(1):65–73. 26053596 10.1002/uog.14919

[46] Donofrio MT , Bremer YA, Schieken RM, et al Autoregulation of cerebral blood flow in fetuses with congenital heart disease: The brain sparing effect. Pediatr Cardiol. 2003;24(5):436–443. 14627309 10.1007/s00246-002-0404-0

[47] McQuillen PS , Goff DA, Licht DJ. Effects of congenital heart disease on brain development. Prog Pediatr Cardiol. 2010;29(2):79–85. 20802830 10.1016/j.ppedcard.2010.06.011 PMC2927012

[48] Kaltman JR , Di H, Tian Z, Rychik J. Impact of congenital heart disease on cerebrovascular blood flow dynamics in the fetus. Ultrasound Obstet Gynecol. 2005;25(1):32–36. 15593334 10.1002/uog.1785

[49] Berg C , Gembruch O, Gembruch U, Geipel A. Doppler indices of the middle cerebral artery in fetuses with cardiac defects theoretically associated with impaired cerebral oxygen delivery in utero: Is there a brain-sparing effect? Ultrasound Obstet Gynecol off J Int Soc Ultrasound Obstet Gynecol. 2009;34(6):666–672. 10.1002/uog.7474 19953563

[50] Szwast A , Tian Z, McCann M, Soffer D, Rychik J. Comparative analysis of cerebrovascular resistance in fetuses with single-ventricle congenital heart disease. Ultrasound Obstet Gynecol. 2012;40(1):62–67. 22407644 10.1002/uog.11147

[51] Loh WY , Anderson PJ, Cheong JLY, et al Neonatal basal ganglia and thalamic volumes: Very preterm birth and 7-year neurodevelopmental outcomes. Pediatr Res. 2017;82(6):970–978. 28700568 10.1038/pr.2017.161 PMC5685902

[52] Ball G , Pazderova L, Chew A, et al Thalamocortical connectivity predicts cognition in children born preterm. Cereb Cortex. 2015;25(11):4310–4318. 25596587 10.1093/cercor/bhu331 PMC4816783

[53] Young JM , Powell TL, Morgan BR, et al Deep grey matter growth predicts neurodevelopmental outcomes in very preterm children. Neuroimage. 2015;111:360–368. 25711136 10.1016/j.neuroimage.2015.02.030

[54] Gertsvolf N , Votava-Smith JK, Ceschin R, et al Association between subcortical morphology and cerebral white matter energy metabolism in neonates with congenital heart disease. Sci Rep. 2018;8(1):14057. 30232359 10.1038/s41598-018-32288-3 PMC6145929

[55] Lim JM , Kingdom T, Saini B, et al Cerebral oxygen delivery is reduced in newborns with congenital heart disease. J Thorac Cardiovasc Surg. 2016;152(4):1095–1103. 27349283 10.1016/j.jtcvs.2016.05.027

[56] Owen M , Shevell M, Donofrio M, et al Brain volume and neurobehavior in newborns with complex congenital heart defects. J Pediatr. 2014;164(5):1121–1127.e1. 24367983 10.1016/j.jpeds.2013.11.033 PMC4474232

[57] Hiraiwa A , Kawasaki Y, Ibuki K, et al Brain development of children with single ventricle physiology or transposition of the great arteries: A longitudinal observation study. Semin Thorac Cardiovasc Surg. 2020;32(4):936–944. 31306764 10.1053/j.semtcvs.2019.06.013

