# Supplementary material for: Individualized brain development and cognitive outcome in infants with congenital heart disease
Source: Brain Commun. 2021 Mar 23;3(2):fcab046. doi: 10.1093/braincomms/fcab046 (PMC8032964; doi:10.1093/braincomms/fcab046)
Supplement: fcab046_Supplementary_Data [file fcab046_supplementary_data.docx]

**Supplementary Materials**

| Supplementary Table 1. CDO_2_ quintile bands | |
| --- | --- |
| Quintile | CDO_2_ measure |
| 1^st^ | 1011-1388 |
| 2^nd^ | 1388-1542 |
| 3^rd^ | 1542-1757 |
| 4^th^ | 1757-2225 |
| 5^th^ | 2225-3023 |

| Supplementary Table 2. Atypicality indices in infants with CHD born ≥37 weeks | | | | | | | |
| --- | --- | --- | --- | --- | --- | --- | --- |
| Region | Whole group Atypicality Index  Mean (SD) | Abnormal streaming of blood  Atypicality Index  Median (IQR) | Left heart abnormalities Atypicality Index  Median (IQR) | Right heart abnormalities Atypicality Index  Median (IQR) | Kruskal wallis H  (p_FDR_) | Extreme negative deviations  Number (%) | Extreme positive deviations  Number (%) |
| Cortical Grey Matter | -0.52 (0.86) | -0.63  (-1.05 - -0.21) | -0.56  (-0.94-0.31) | -0.57  (-1.26 - 0.19) | 0.88 (0.64) | 0 (0) | 0 (0) |
| White Matter | -0.54 (0.97) | -0.87  (-1.25 - -0.10) | -0.51  (-0.99-0.40) | -0.76  (-1.18- 0.01) | 1.64 (0.52) | 0 (0) | 0 (0) |
| Extracerebral CSF | 0.66 (1.19) | 0.48  (0.09-1.05) | 0.79  (0.14-1.59) | -0.70  (-0.64-1.03) | 1.69 (0.56) | 0 (0) | 5 (8) |
| Ventricles  median (IQR) | 0.01  (-0.76-1.19) | 0.13  (0.80-1.19) | 0.22  (-0.12 -1.20) | -0.55  (-1.07-0.39) | 5.16  (0.31) | 0 (0) | 2 (3) |
| Cerebellum | -0.37 (1.01) | -0.41  (-0.85-0.05) | 0.19  (-1.05 -0.63) | -0.32  (-0.74- 0.23) | 2.18 (0.42) | 1 (2) | 0 (0) |
| Brainstem | -0.52 (1.10) | -0.88  (-1.31 - -0.39) | -0.27  (-1.07 -0.40) | -0.43  (-0.92 -0.48) | 2.99 (0.48) | 1 (2) | 0 (0) |
| Left Thalamus | -0.52 (0.93) | -0.71  (-1.48- -0.21) | -0.26  (-0.52-0.56) | -0.20  (-1.20-0.04) | 4.22 (0.31) | 0 (0) | 0 (0) |
| Right Thalamus | -0.66 (0.92) | -0.97  (-1.40- -0.13) | -0.43  (-0.94-0.23) | -0.16  (-0.61- -0.07) | 1.92 (0.52) | 0 (0) | 0 (0) |
| Left Lentiform | -0.47 (0.85) | -0.63  (-1.15- -0.26) | -0.39  (-0.68-0.29) | -0.68  (-1.06- -0.02) | 2.57 (0.52) | 0 (0) | 0 (0) |
| Right Lentiform | -0.11 (0.94) | -0.33  (-0.88-0.21) | 0.40  (-0.34-0.71) | -0.16  (-1.04-0.01) | 4.59 (0.31) | 0 (0) | 0 (0) |
| Left Caudate Nucleus | -0.64 (0.94) | -1.24  (-1.80- -0.21) | -0.34  (-0.71-0.22) | -0.21  (-0.71- -0.13) | 6.21 (0.31) | 0 (0) | 0 (0) |
| Right Caudate Nucleus | -0.67 (0.95) | -0.98  (-1.72- -0.16) | -0.17  (-0.75-0.32) | -0.44  (-0.98- -0.33) | 5.17 (0.31) | 0 (0) | 0 (0) |
| Total tissue volume | -0.56 (0.91) | -0.84  (-1.11- -0.29) | -0.47  (-0.96-0.38) | -0.64  (-1.50- 0.21) | 1.31 (0.52) | 0 (0) | 0 (0) |

| Supplementary Table 3. Clinical information for infants with extreme deviations in intracranial volumes. | | | | | | | |
| --- | --- | --- | --- | --- | --- | --- | --- |
| Sex | GA at birth | PMA at scan | Birth weight z-score | Diagnosis | Brain Injury Findings | CDO_2_  ml O_2_/min (quintile) | Extreme deviations  affected regions (atypicality index) |
| M | 34+6 | 37+4 | -0.22 | Tricuspid Atresia with additional abnormalities | Severe WMI | 1011 (1) | Brainstem (-2.98)  Left Caudate  (-3.07)  Right Caudate  (-2.66)  Left Thalamus  (-3.03)  Total Tissue Volume (-2.69) |
| F | 38+3 | 39+2 | -1.01 | TGA | Mild WMI | 1594 (3) | Brainstem (-3.30) |
| M | 38+5 | 39+5 | -0.94 | Tricuspid atresia | None | 1168 (1) | Cerebellum (-2.86) |
| F | 38+2 | 39+1 | -2.25 | CoA | Mild WMI | x | Ventricles (4.52) |
| M | 38+2 | 39+2 | 0.99 | TGA | None | 2355 (5) | Extracerebral CSF (2.74)  Ventricles (2.76) |
| F | 38+2 | 38+2 | 0.79 | HLHS | None | x | Extracerebral CSF (3.89) |
| M | 38+6 | 39+3 | 1.49 | CoA | Cerebellar Haemorrhage | 3023 (5) | Extracerebral CSF (3.49) |
| M | 38+1 | 39+0 | -0.37 | TGA | None | 1650 (3) | Extracerebral CSF (2.77) |
| F | 39+0 | 39+5 | -0.22 | CoA | Moderate WMI | x | Extracerebral CSF (2.73) |
| Gestational Age, GA; Postmenstrual Age, PMA; Cerebral Oxygen Delivery, CDO_2_; Female, F; Male, M; dextro-transposition of the great arteries, TGA; Hypoplastic left heart syndrome, HLHS; Coarctation of the Aorta, CoA; White Matter Injury, WMI. | | | | | | | |

| Supplementary Table 4. Correlations between atypicality indices and Index of Multiple Deprivation across the whole sample | | |
| --- | --- | --- |
| Region | ρ | p_FDR_ |
| Left Thalamus | 0.35 | 0.23 |
| Right Thalamus | 0.31 | 0.23 |
| Left Caudate | 0.18 | 0.38 |
| Right Caudate | 0.09 | 0.60 |
| Cortical Grey Matter | 0.25 | 0.32 |
| White Matter | 0.21 | 0.32 |
| Cerebellum | 0.21 | 0.32 |
| Brainstem | 0.21 | 0.32 |
| Left Lentiform | 0.03 | 0.83 |
| Right Lentiform | 0.14 | 0.54 |
| Total Tissue Volume | 0.30 | 0.23 |
| Extracerebral CSF | 0.09 | 0.60 |
| Ventricles | 0.12 | 0.56 |

| Supplementary Table 5. Regression coefficients for models predicting Cognitive Composite score in infants with CHD born ≥37 weeks | | |
| --- | --- | --- |
| Variable | β | p |
| **Cognitive Composite Score ~ Left Thalamus Atypicality Index + Index of Multiple Deprivation** | | |
| Left Thalamus Atypicality Index | 0.53 | <0.001 |
| Index of Multiple Deprivation | -0.53 | <0.001 |
| *R^2^ [95% CI] = 0.33 [0.08-0.53]; adjusted R^2^ = 0.30; F(2,38) = 9.40, p_FDR_ = 0.004* | | |
| **Cognitive Composite Score ~ Right Thalamus Atypicality Index + Index of Multiple Deprivation** | | |
| Right Thalamus Atypicality Index | 0.48 | 0.003 |
| Index of Multiple Deprivation | -0.51 | 0.002 |
| *R^2^ [95% CI] = 0.29 [0.06-0.51]; adjusted R^2^ = 0.25; F(2,38) = 7.62, p_FDR_ = 0.004* | | |
| **Cognitive Composite Score ~ Left Caudate Nucleus Atypicality Index + Index of Multiple Deprivation** | | |
| Left Caudate nucleus Atypicality Index | 0.38 | 0.007 |
| Index of Multiple Deprivation | -0.41 | 0.007 |
| *R^2^ [95% CI] = 0.26 [0.04-0.44]; adjusted R^2^ = 0.22; F(2,38) = 6.74, p_FDR_ = 0.005* | | |
| **Cognitive Composite Score ~ Right Caudate Nucleus Atypicality Index + Index of Multiple Deprivation** | | |
| Right Caudate nucleus Atypicality Index | 0.41 | 0.004 |
| Index of Multiple Deprivation | -0.37 | 0.012 |
| *R^2^ [95% CI] = 0.28 [0.07-0.44]; adjusted R^2^ = 0.25; F(2,38) = 7.52, p_FDR_ = 0.005* | | |
| **Cognitive Composite Score ~ Left Lentiform nucleus Atypicality Index + Index of Multiple Deprivation** | | |
| Left Lentiform nucleus Atypicality Index | 0.44 | 0.009 |
| Index of Multiple Deprivation | -0.34 | 0.022 |
| *R^2^ [95% CI] = 0.25 [0.04-0.45]; adjusted R^2^ = 0.21; F(2,38) =6.47, p_FDR_ = 0.005* | | |
| **Cognitive Composite Score ~ Cortical Grey Matter Atypicality Index + Index of Multiple Deprivation** | | |
| Cortical Grey Matter Atypicality Index | *0.43* | *0.016* |
| Index of Multiple Deprivation | *-0.42* | *0.018* |
| *R^2^ [95% CI] = 0.21 [0.03-0.41]; adjusted R^2^ = 0.16; F(2,36) = 4.66, p_FDR_ = 0.019* | | |
| **Cognitive Composite Score ~ Total Tissue Volume Atypicality Index + Index of Multiple Deprivation** | | |
| Total Tissue Volume Atypicality Index | *0.41* | *0.021* |
| Index of Multiple Deprivation | *-0.41* | *0.22* |
| *R^2^ [95% CI] = 0.19 [0.02-0.41]; adjusted R^2^ = 0.15; F(2,38) = 4.34, p_FDR_ = 0.021* | | |

| Supplementary Table 6. Correlation between CDO_2_ and brain volume atypicality indices | | |
| --- | --- | --- |
| Region | Correlation with CDO_2_ | |
|  | Whole Sample  r (p_FDR_) | Infants born ≥37 weeks  r (p_FDR_) |
| Cortical Grey Matter | 0.42 (0.038) | 0.37 (0.030) |
| White Matter | 0.38 (0.017) | 0.32 (0.051) |
| Cerebellum | 0.30 (0.047) | 0.25 (0.11) |
| Brainstem | 0.38 (0.015) | 0.32 (0.049) |
| Left Thalamus | 0.42 (0.008) | 0.36 (0.030) |
| Right Thalamus | 0.33 (0.034) | 0.27 (0.089) |
| Left Caudate | 0.29 (0.047) | 0.24 (0.11) |
| Right Caudate | 0.30 (0.041) | 0.24 (0.11) |
| Left Lentiform | 0.41 (0.008) | 0.37 (0.03) |
| Right Lentiform | 0.43 (0.008) | 0.38 (0.03) |
| Total tissue volume | 0.42 (0.008) | 0.37 (0.03) |
| Ventricles | 0.08 (0.56) | 0.05 (0.73) |
| Extracerebral CSF | 0.11 (0.48) | 0.05 (0.73) |

**
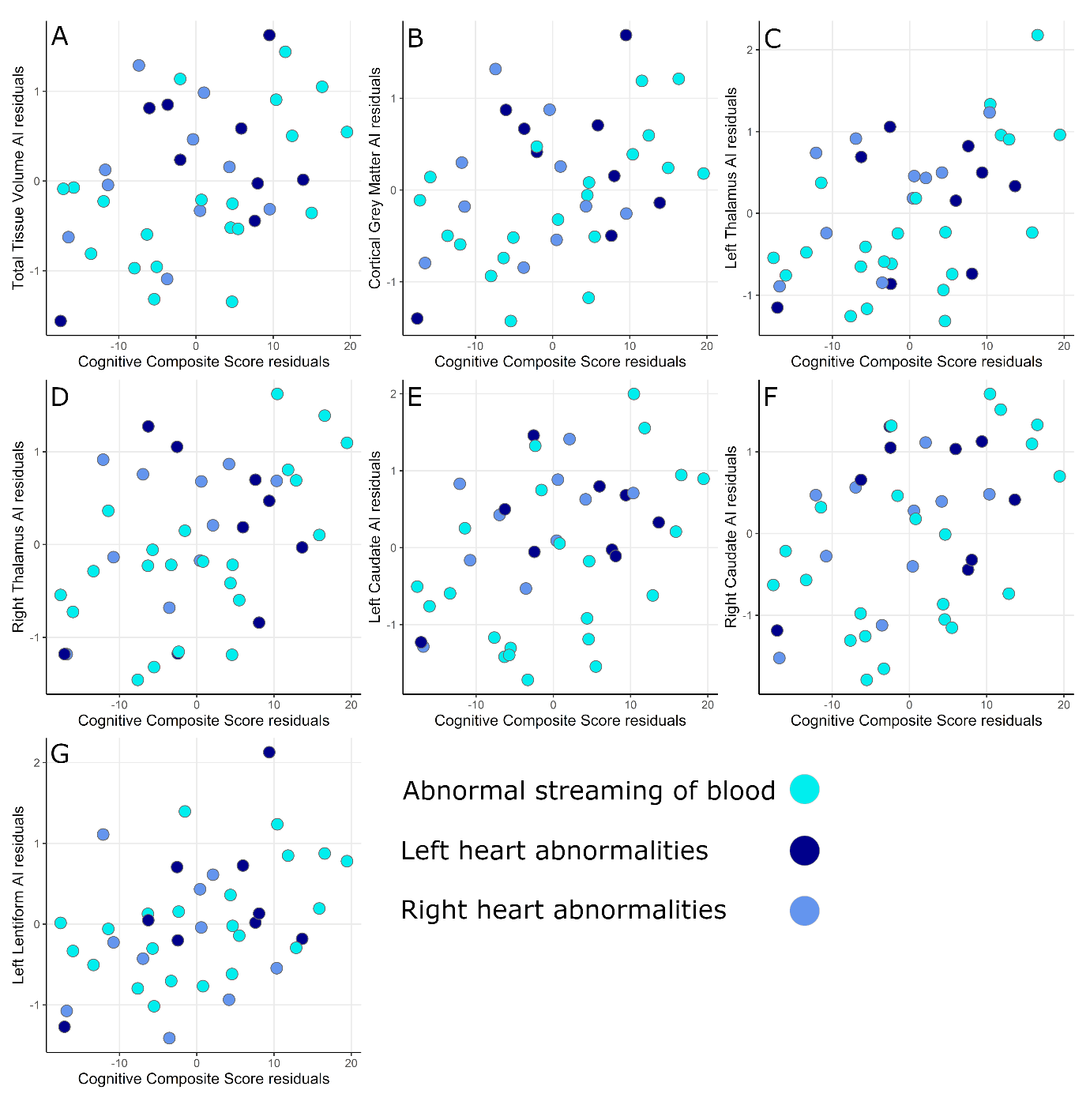
**

**Supplementary Figure 1. Associations between cognitive scores and atypicality indices in infants born ≥37 weeks.** Scatter plots showing cognitive composite score residuals plotted against (A) total tissue volume (B) cortical grey matter (C) left thalamus (D) right thalamus (E) left caudate (F) right caudate (G) left lentiform atypicality index infants with CHD born ≥37 weeks. Residuals are corrected for index of multiple deprivation. AI=Atypicality Index.

**
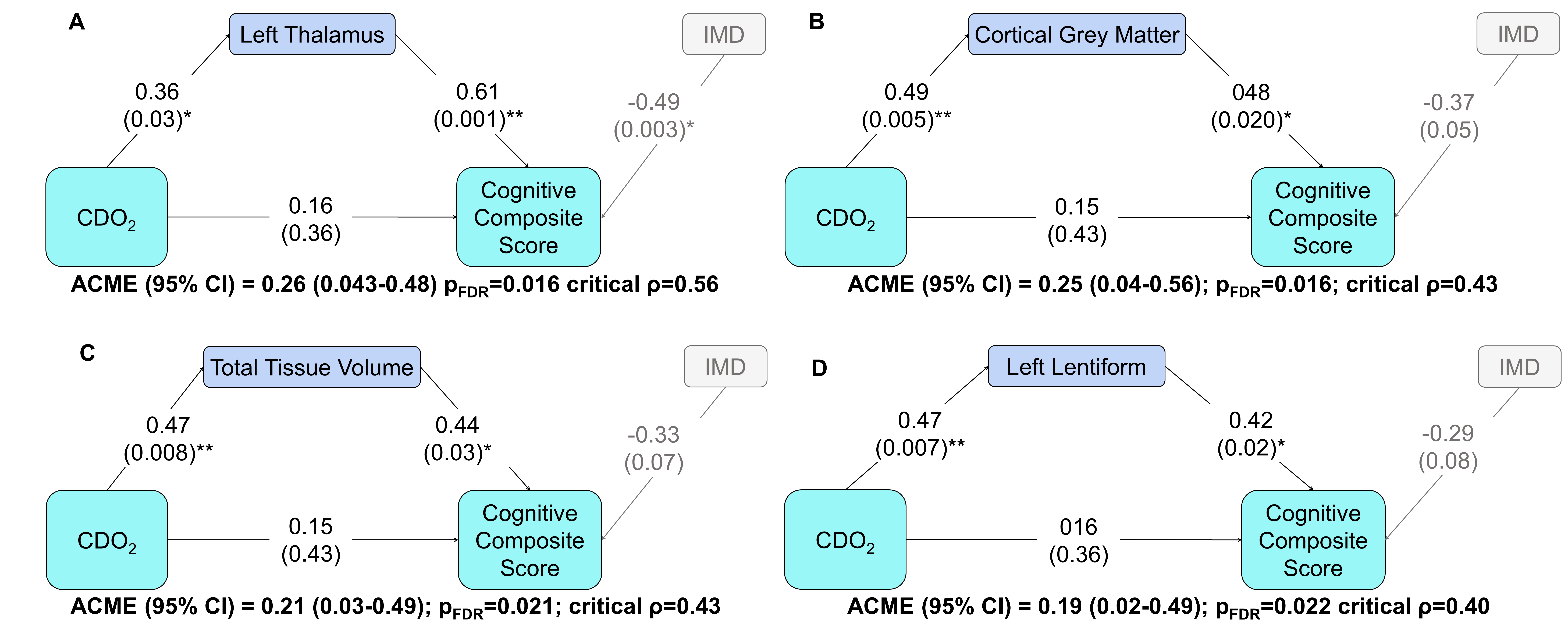
**

**Supplementary Figure 2.** **The relationship between CDO_2_ and Cognitive Composite Scores mediated by atypicality indices in infants born ≥37 weeks.**  Path diagrams showing the indirect relationship between CDO_2_ and cognitive composite score mediated by (A) left thalamus (B) cortical grey matter (C) total tissue volume (D) left lentiform atypicality indices in infants born ≥37 weeks. Standardised regression coefficients are reported; numbers in brackets show p-value *p<0.05 **p<0.01. ACME=Average Causal Mediation Effect; SCDO_2_=Cerebral Oxygen Delivery.
